# First record of *Doleschallia tongana* (Lepidoptera: Nymphalidae) for Guam Island

**DOI:** 10.12688/f1000research.14316.1

**Published:** 2018-03-23

**Authors:** Jake Manuel, W. John Tennent, Donald W. Buden, Aubrey Moore

**Affiliations:** 1 College of Natural and Applied Sciences, University of Guam, Mangilao, Guam, 96923, USA; 2 Natural History Museum, London, SW7 5BD, UK; 3 Division of Natural Sciences and Mathematics, College of Micronesia-FSM, Kolonia, Pohnpei, 96941, Federated States of Micronesia

**Keywords:** Doleschallia tongana, Pacific orange leafwing, Guam, Micronesia, range expansion, invasive species, new country record

## Abstract

A single specimen of the butterfly, *Doleshallia tongana* Hopkins 1927, was collected on Guam Island on October 23, 2017 (13.430478°N, 144.800419°E). This is a new species record for Guam and Micronesia, indicating a geographical range expansion for *D. tongana*.

## Introduction

On October 23, 2017, a butterfly was taken from the underside of a leaf of soursop, *Annona muricata*, by a student (JM) assembling an insect collection as a requirement for the General Entomology course at the University of Guam. The collection site was the University of Guam campus in Mangilao, Guam (13.430478° N, 144.800419° E).

**Figure 1.**
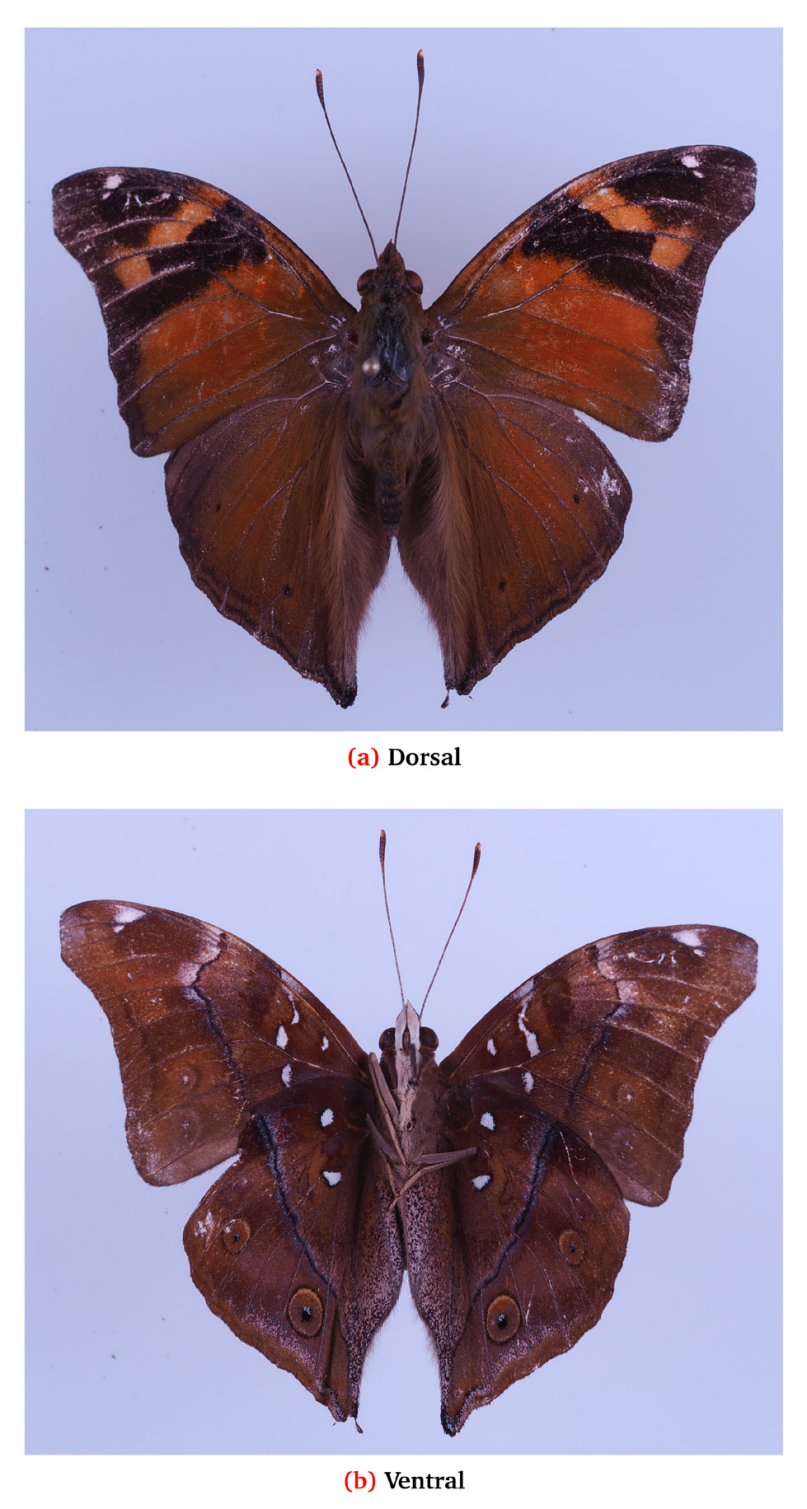
First specimen of *Doleschallia tongana* collected on Guam.

The specimen was pinned, images were made ( Figure 1), documented in iNaturalist ^1^ and deposited in the University of Guam insect collection (Accession code: iNat8515898).

This specimen does not match any of the descriptions in *Butterflies of Micronesia* ^2^, the standard reference for Guam’s butterflies.

## Identification

Digital images of the specimen were sent to DB and JT for identification. On 7 November 2017, DB tentatively identified the specimen as a species in the genus *Doleschallia*, and indicated it possibly belonging to the *bisaltide* complex. On 24 February, 2018 JT determined the butterfly as *Doleschallia tongana* Hopkins, 1927, based on images and comparison with the extensive collections of the Natural History Museum, London.

In common with other species in the “ *bisaltide* species-group, *D. tongana* is individually variable.

The convex outer margin of the forewing; the general appearance of the specimen; and geography all suggest *D. tongana* ( *tongana* Hopkins, 1927, is a name to replace *drusias* Fabricius, 1781, the type locality for which is Tonga). Some minor ‘unusual’ features include the fact that *tongana* usually has a sub-apical ‘half-moon’ series of 4–5 spots on the forewing, lacking in this specimen, which only has two, but this lies within the wide individual variation of the species. Considering a distribution of Papua New Guinea (including the Bismarcks), the Solomon Islands, Fiji, Samoa, Tonga and New Caledonia, we are confident of associating this specimen with *D. tongana*. No doubt further material will confirm this association in due course. The species-group is in need of some revision ^3^. The GBIF Backbone Taxonomy lists the accepted name for this taxon as *Doleschallia bisaltide* subsp. *tongana* Hopkins, 1927 ^4^. However, the taxon record is tagged as a "name parent mismatch" issue.

*D. tongana* is listed in the iNaturalist database ^5^ and has been assigned the vernacular name ’Pacific orange leafwing’.

## Geographical distribution

*D. tongana*, as it is currently understood, occurs throughout much of New Guinea, including the island groups in the east (see above).

Occurrence of *D. tongana* in Samoa is a relatively recently recorded range expansion. It was first detected on Tutuila Island in American Samoa in 1997 ^6^. Cook and Vargo 2000 ^6^ state that "The inclusion of Samoa in this species’ range by Parsons, 1998 ^7^ appears to be based on a misreading of Hopkins (1927)."

## Description of caterpillar

Cook and Vargo 2000 ^6^ provide a description of a last instar *D. tongana* caterpillar:

“Just prior to pupation, the caterpillar measured ca. 50 mm in length. It possessed a black ground color with light speckling dorsally and prominent cream colored stripes running longitudinally, located dorso-laterally and ventro-laterally. Each body segment had seven prominent black spines, with numerous smaller secondary spines. The base of each primary spine was pale metallic blue. From a distance, the most prominent features of the caterpillar are the black ground color with metallic blue spots, and the pair of light parallel stripes running longitudinally on each side.“

Only a few larval host plants have been recorded for *D.tongana* ( Table 1).

**Table 1.** Larval host plants of *Doleschallia tongana*.

Larval host plant	Reference(s)
Acanthaceae	
* Graptophyllum*	
* Graptophyllum insularum*	8
* Graptophyllum pictum*	6, 7
* Pseuderanthemum*	
* Pseuderanthemum carruther*	6
* Pseuderanthemum laxiflorum*	8
* Pseuderanthemum* sp.	9
Moraceae	
* Artocarpus*	
* Artocarpus altilis*	8
Fabaceae	
* Erythrina*	
* Erythrina* sp.	8

## Discussion

An informal survey has been initiated on Guam to search for more specimens of *D. tongana* and to record host plants.

This insect has the potential to do economic damage because it has been reported to feed on breadfruit, *Artocarpus altilis* ^8^.

## Data availability

All data underlying the results are available as part of the article and no additional source data are required.
