# Supplementary material for: First record of Doleschallia tongana (Lepidoptera: Nymphalidae) for Guam Island
Source: F1000Res. 2018 Mar 23;7:366. [Version 1] doi: 10.12688/f1000research.14316.1 (PMC5968358; doi:10.12688/f1000research.14316.1)
Supplement: Peer review history

# Peer review: Referee response for version 1

**DOI:** 10.5256/f1000research.15578.r33973

**Published:** 2018-05-23

**Authors:** Niklas Wahlberg

**Affiliations:** 1 Department of Biology, Lund University, Lund, Sweden

This is a simple report on a range extension of a butterfly species. What makes it interesting is that the range extension is to the island of Guam, which is relatively isolated. It seems this species is on a rampage through the South Pacific, colonizing new isolated islands! The only reference I missed was the relatively recent book by Patrick and Patrick (2012) "Butterflies of the South Pacific". I unfortunately do not have access to my copy at the moment to see what it says about the species. Perhaps good to look at that?

I have read this submission. I believe that I have an appropriate level of expertise to confirm that it is of an acceptable scientific standard.

# Peer review: Referee response for version 1

**DOI:** 10.5256/f1000research.15578.r32407

**Published:** 2018-03-28

**Authors:** Richard S. Zack

**Affiliations:** 1 Department of Entomology, Washington State University, Pullman, WA, USA

A well-written report and discussion of a new species of butterfly to Guam.  The record is a significant range extension for the species.  Introductions to islands such as Guam are of concern because of potential economic and environmental effects and reports such as this are important to document.  It will be interesting to see if the species has established or if this was a one-time occurrence.

I have read this submission. I believe that I have an appropriate level of expertise to confirm that it is of an acceptable scientific standard.
